# Pembrolizumab with platinum-based chemotherapy with or without epacadostat as first-line treatment for metastatic non-small cell lung cancer: a randomized, partially double-blind, placebo-controlled phase II study

**DOI:** 10.1186/s12885-022-10427-4

**Published:** 2024-07-25

**Authors:** Michael Boyer, Rina Hui, Damien Urban, Philip Clingan, Wu-Chou Su, Celine Devaux, Shirish Gadgeel, Marina Garassino, Lance Leopold, Jeannie Daniel, Mihaela C. Munteanu, Ayman Samkari, Yiwen Luo, Delvys Rodriguez Abreu

**Affiliations:** 1 Chris O’Brien Lifehouse, University of Sydney, Missenden Road, PO BOX M33, Camperdown, NSW 2050 Australia; 2 Crown Princess Mary Cancer Centre, Westmead Hospital, University of Sydney, Westmead, NSW Australia; 3 Department of Oncology, Chaim Sheba Medical Center, Tel Aviv University Sackler School of Medicine, Ramat Gan, Israel; 4 Southern Medical Day Care Centre, Wollongong, NSW Australia; 5 Department of Oncology, National Cheng Kung University Hospital, Tainan, Taiwan; 6 Centre Intégré de Santé Et de Services Sociaux de La Montérégie-Centre, Greenfield Park, QC Canada; 7 Department of Internal Medicine, Division of Hematology/Oncology, University of Michigan, Ann Arbor, MI USA; 8 Department of Medicine, University of Chicago, Chicago, IL USA; 9 Incyte Corporation, Wilmington, DE USA; 10 Merck & Co., Inc., Rahway, NJ USA; 11 Department of Medical Oncology, Complejo Hospitalario Universitario Insular-Materno Infantil. Universidad de Las Palmas de Gran Canaria, Gran Canaria, Spain

**Keywords:** Epacadostat, Combination immunotherapy, Non-small cell lung cancer, Pembrolizumab

## Abstract

**Background:**

The combination of the checkpoint inhibitor (CPI) pembrolizumab and platinum-based chemotherapy is effective frontline therapy for advanced non-small cell lung cancer (NSCLC) lacking targetable mutations. Indoleamine 2,3- dioxygenase 1 (IDO1), an enzyme involved in kynurenine production, inhibits immune responses. Inhibition of IDO1 may restore antitumor immunity and augment CPI activity. This trial evaluated addition of epacadostat, a potent and highly selective IDO1 inhibitor, to pembrolizumab and chemotherapy for metastatic NSCLC.

**Methods:**

ECHO-306/KEYNOTE-715 was a partial double-blind, randomized phase II study of adults with treatment-naïve stage IV NSCLC not indicated for EGFR-, ALK-, or ROS1-directed therapy. Patients were randomized to one of three treatment arms: epacadostat-pembrolizumab-chemotherapy (E + P + C; blinded), epacadostat-pembrolizumab (E + P; open-label) or placebo-pembrolizumab-chemotherapy (PBO + P + C; blinded). Stratification was by PD-L1 tumor proportion score (< 50% vs. ≥ 50%) and tumor histology (non-squamous vs. squamous). A protocol amendment closed enrollment in the open-label E + P group, excluding it from efficacy analyses. Intravenous pembrolizumab (200 mg) was administered every 21 days and epacadostat 100 mg or matching placebo (oral) twice daily (BID) for ≤ 35 3-week cycles. The primary objective was objective response rate (ORR) for E + P + C vs. PBO + P + C.

**Results:**

178 patients were randomized to E + P + C (*n* = 91) or PBO + P + C (*n* = 87); 55 were enrolled in the E + P group. The E + P + C group had a lower confirmed ORR (26.4%; 95% CI 17.7–36.7) than the PBO + P + C group (44.8%; 95% CI 34.1–55.9), with a difference of − 18.5% (95% CI − 32.0 – (− 4.3); one-sided *P* = 0.9948). The E + P + C group had a numerically higher percentage of confirmed responders with extended response ≥ 6 months (29.2% vs. 15.4%). Circulating kynurenine levels at C1D1 were similar to those at C2D1 in all treatment groups and were not reduced to normal levels with epacadostat 100 mg BID plus P + C. The safety profile of E + P + C was consistent with that for PBO + P + C.

**Conclusions:**

Addition of epacadostat 100 mg BID to pembrolizumab and platinum-based chemotherapy was generally well tolerated but did not improve ORR in patients with treatment-naïve metastatic NSCLC. Evaluating epacadostat doses that normalize circulating kynurenine in combination with CPIs may help determine the clinical potential of this combination.

**Trial registration:**

NCT03322566. Registered October 26, 2017.

**Supplementary Information:**

The online version contains supplementary material available at 10.1186/s12885-022-10427-4.

## Background

Non-small cell lung cancer (NSCLC) represents ~ 84% of all lung cancers [1]. The majority of patients have metastatic disease at diagnosis [1, 2]. Chemotherapy combined with pembrolizumab, an immune checkpoint inhibitor that inhibits programmed cell death protein 1 (PD-1), is an efficacious option for first-line treatment of advanced NSCLC lacking targetable mutations [3, 4]. However, NSCLC cells evade immune surveillance via multiple mechanisms, so approaches targeting multiple immune pathways may provide enhanced efficacy in this setting.

Indoleamine 2,3-dioxygenase 1 (IDO1), an enzyme that catalyzes the breakdown of tryptophan to kynurenine, is constitutively expressed by a wide variety of human tumor cell types as well as by dendritic cells that localize to tumor-draining lymph nodes [5, 6]. Some NSCLC tumors co-express programmed death-ligand 1 (PD-L1) and IDO1 [7–9]. Overexpression of IDO1 has been shown to result in suppression of T cell-mediated immune responses [5, 10]*.* IDO1, which is induced by interferon, can also suppress inflammatory responses targeting tumor cells [5, 11–14]*.* Thus, inhibition of IDO1 may help restore an effective antitumor response and may also augment checkpoint inhibitor activity by reducing resistance to anti-tumor inflammatory responses.

Epacadostat is a potent selective inhibitor of IDO1 [15, 16] that has been shown to normalize plasma kynurenine levels in patients with advanced solid tumors at twice-daily (BID) doses ≥ 100 mg as monotherapy [16]. The combination of epacadostat and pembrolizumab has been investigated in NSCLC and other types of advanced solid tumors. In the phase I/II ECHO-202/KEYNOTE-037 study of epacadostat plus pembrolizumab in patients with advanced tumors, promising responses were observed in melanoma (ORR 60.5%) and NSCLC with PD-L1 tumor proportion score (TPS) < 50% (ORR 24.4%), and treatment was generally well tolerated [17]. We present results from the final analysis of a phase II randomized study (NCT03322566) assessing the safety and efficacy of epacadostat plus pembrolizumab with platinum-based chemotherapy (E + P + C) vs. placebo plus pembrolizumab with platinum-based chemotherapy (PBO + P + C) in patients with metastatic NSCLC.

## Methods

### Study design and conduct

ECHO-306/KEYNOTE-715 was an active comparator, partial double-blind, parallel-group, multicenter randomized phase II study. The study was originally planned as a phase III study. However, after the study was initiated, it was amended to a phase II study design by a protocol amendment based on data from the ECHO-301/KEYNOTE-252 study in unresectable/metastatic melanoma that showed that epacadostat 100 mg BID in combination with pembrolizumab did not improve the primary endpoint of progression-free survival (PFS) compared with pembrolizumab monotherapy [18]. Other changes in study methods based on this protocol amendment are described in their respective sections below.

This study was conducted in accordance with the Declaration of Helsinki, Good Clinical Practice guidelines, applicable country and/or local statutes, regulations regarding independent ethics committee review, and the protection of human patients in biomedical research. All patients provided written informed consent before initiating treatment.

### Study population

Adults ≥ 18 years of age with confirmed stage IV NSCLC not indicated for EGFR-, ALK-, or ROS1-directed therapy, measurable disease per Response Evaluation Criteria in Solid Tumors version 1.1 (RECIST v1.1), life expectancy ≥ 3 months, Eastern Cooperative Oncology Group (ECOG) performance status 0 or 1 and adequate organ function based on laboratory values were eligible for enrollment. Exclusion criteria included prior systemic chemotherapy or other targeted/biological antineoplastic therapy for metastatic NSCLC; prior treatment with any anti-PD-1, anti-PD-L1, anti-PD-L2 or anti-IDO1 agent or with an agent directed to another stimulatory or co-inhibitory T-cell receptor; radiotherapy ≤ 14 days or lung radiation therapy > 30 Gy ≤ 6 months before the first study treatment dose; systemic steroid therapy ≤ 7 days prior to the first dose of study treatment or any other form of immunosuppressive medication; history of serotonin syndrome after receiving serotonergic drugs; history of (non-infectious) pneumonitis requiring systemic steroids or current pneumonitis/interstitial lung disease and untreated central nervous system (CNS) metastases and/or carcinomatous meningitis. Tumor PD-L1 expression levels were determined using the PD-L1 IHC 22C3 pharmDx assay (Agilent Technologies, Carpinteria, CA).

### Study procedure and interventions

In the original design, patients were randomized to the E + P + C, PBO + P + C or open-label E + P treatment arms and stratified by PD-L1 TPS (< 50% vs. ≥ 50%) and tumor histology (squamous vs. non-squamous). In the original study design, enrollment of 1062 patients was planned. The open-label E + P group was closed by protocol amendment 05, which also changed the design to a phase II study, and the target was reduced to 148 planned randomized patients into the existing two blinded treatment arms (E + P + C and PBO + P + C). Patients already randomized to the E + P group at amendment 5 were allowed to continue study treatment if they were deriving clinical benefit, but were not included in the primary efficacy analysis. The efficacy analysis population consisted of all patients randomized to a blinded study intervention (i.e., the intent-to-treat population), and the safety analysis population consisted of all patients as treated who received ≥ 1 dose of study intervention.

Pembrolizumab 200 mg was administered intravenously (IV) every 21 days (Q3W) and epacadostat 100 mg or matching placebo orally BID for up to 35 3-week cycles. Epacadostat could be reduced to 50 or 25 mg BID to mitigate immune-related adverse events (AEs). Platinum-based chemotherapy consisted of one of three regimens selected by the investigator prior to randomization. For non-squamous tumor histology, pemetrexed 500 mg/m^2^ IV with either cisplatin 75 mg/m^2^ IV or carboplatin area under the curve (AUC) 5 mg/mL/min IV was given Q3W for four cycles, followed by pemetrexed 500 mg/m^2^ IV Q3W for up to 35 cycles. For squamous tumor histology, paclitaxel 175 − 200 mg/m^2^ IV + carboplatin AUC 5 − 6 mg/mL/min IV was given Q3W for four cycles.

Fasted patients had blood drawn before dosing on day 1 of cycle 1 (C1D1) and day 1 of cycle 2 (C2D1). Serum kynurenine levels were determined by a proprietary, validated liquid chromatography-tandem mass spectrometry assay using calibrated standards at Worldwide Clinical Trials, Morrisville, NC.

### Study objectives and endpoints

The original primary endpoints were overall survival (OS) and PFS. The primary endpoint was changed to ORR when a protocol amendment changed the study from a phase III to a phase II study, with the primary objective of the study comparing the ORR of combination E + P + C versus PBO + P + C. ORR was defined per RECIST v1.1 based on confirmed response by blinded independent central review (BICR). The secondary objectives were to compare PFS, OS, duration of response (DOR) and safety/tolerability of E + P + C versus PBO + P + C. AEs were graded according to the National Cancer Institute Common Terminology Criteria for Adverse Events (NCI CTCAE) version 4.0. Assessment of pharmacodynamic activity of epacadostat based on changes in circulating kynurenine levels from baseline was among the exploratory objectives.

### Statistical analyses

ORR was compared using the Miettinen and Nurminen method [19] stratified by PD-L1 TPS (< 50% vs ≥ 50%) and tumor histology (squamous vs non-squamous). Because of the small sample size, the strata ‘PD-L1 TPS ≥ 50 percent non-squamous’ and ‘PD-L1 TPS ≥ 50 percent squamous’ were combined into one stratum for the primary analysis. Based on the assumption of 74 patients randomized per arm with at least 12 weeks of follow-up, the study had 81.7% power to detect a clinically meaningful (ie, 20 percentage-point difference) in ORR between the E + P + C and PBO + P + C groups at α = 5% (one-sided). PFS and OS were compared using a stratified log-rank test. Hazard ratios (HRs) were estimated using a stratified Cox regression model with Efron’s method of tie handling. Median PFS, median OS, PFS rates and OS rates were estimated with the Kaplan–Meier method. Circulating kynurenine levels within each treatment group were compared using paired t-tests.

## Results

### Patient characteristics

**Fig. 1:**
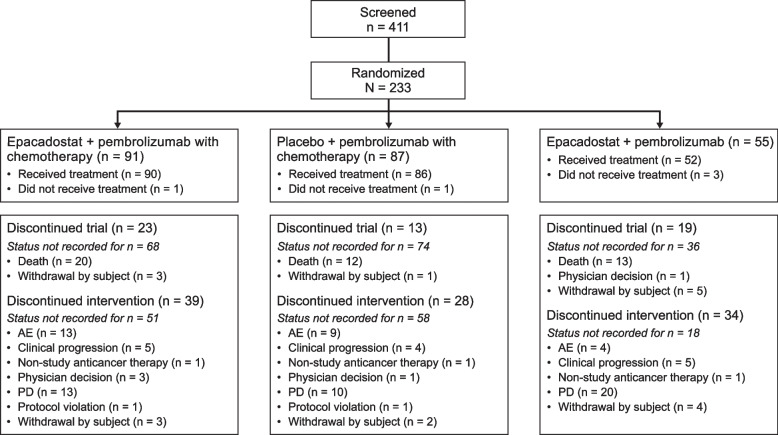
Patient disposition *AE* adverse event; *PD* progressive disease

This study was conducted from January 09, 2018—October 16, 2020. A total of 411 patients were screened and 233 met inclusion criteria and were randomized to E + P + C (*n* = 91), PBO + P + C (*n* = 87) or E + P (*n* = 55) (Fig. 1). The E + P + C and PBO + P + C treatment groups were generally well balanced; more patients had stage M1C tumors in the E + P + C group, and more patients had stage M1B tumors in the PBO + P + C group (> 10% difference) (Table 1). Most patients in both groups were former smokers with predominantly non-squamous tumor histology, PD-L1 TPS < 50% and did not have baseline brain metastasis.

**Table 1:** Patient and disease characteristics

	Epacadostat + pembrolizumab with chemotherapy (*n* = 91)	Placebo + pembrolizumab with chemotherapy (*n* = 87)	Epacadostat + pembrolizumab^a^ (*n* = 55)
Gender			
Male	58 (63.7)	57 (65.5)	39 (70.9)
Female	33 (36.3)	30 (34.5)	16 (29.1)
Age, Years			
< 65	49 (53.8)	46 (52.9)	34 (61.8)
≥ 65	42 (46.2)	41 (47.1)	21 (38.2)
Median (range)	63.0 (31–84)	64.0 (37–82)	63.0 (42–84)
Race			
American Indian or Alaska Native	1 (1.1)	0	0
Asian	11 (12.1)	10 (11.5)	2 (3.6)
Black or African American	1 (1.1)	1 (1.1)	0
White	78 (85.7)	75 (86.2)	53 (96.4)
Missing	0	1 (1.1)	0
Ethnicity			
Hispanic or Latino	3 (3.3)	1 (1.1)	1 (1.8)
Not Hispanic or Latino	86 (94.5)	85 (97.7)	52 (94.5)
Not reported	0	0	1 (1.8)
Unknown	2 (2.2)	1 (1.1)	1 (1.8)
Chemotherapy received			
Carboplatin/paclitaxel	27 (29.7)	23 (26.4)	0
Cisplatin/pemetrexed	6 (6.6)	7 (8.0)	0
Carboplatin/pemetrexed	57 (62.6)	56 (64.4)	0
Missing/no chemotherapy received	1 (1.1)	1 (1.1)	55 (100)
Smoking status			
Never	17 (18.7)	12 (13.8)	6 (10.9)
Former	52 (57.1)	55 (63.2)	36 (65.5)
Current	22 (24.2)	20 (23.0)	13 (23.6)
ECOG performance status			
0	35 (38.5)	27 (31.0)	26 (47.3)
1	55 (60.4)	60 (69.0)	29 (52.7)
2	1 (1.1)	0	0
Predominant tumor histology			
Squamous	26 (28.6)	22 (25.3)	15 (27.3)
Non-squamous^b^	65 (71.4)	65 (74.7)	40 (72.7)
PD-L1 status			
≥ 50%	19 (20.9)	21 (24.1)	8 (14.5)
< 50%	72 (79.1)	66 (75.9)	47 (85.5)
Metastatic stage			
M1	1 (1.1)	0	0
M1A	30 (33.0)	29 (33.3)	17 (30.9)
M1B	15 (16.5)	25 (28.7)	18 (32.7)
M1C	45 (49.5)	33 (37.9)	20 (36.4)
Brain metastasis status at baseline	10 (11.0)	11 (12.6)	7 (12.7)
Prior adjuvant therapy	4 (4.4)	1 (1.1)	5 (9.1)
Prior neo-adjuvant therapy	2 (2.2)	2 (2.3)	2 (3.6)
Prior radiation	26 (28.6)	25 (28.7)	14 (25.5)

Data are *n* (%) unless otherwise noted

*ECOG* Eastern cooperative oncology group, *PD-L1* Programmed death-ligand 1, *SD* Standard deviation

^a^The epacadostat + pembrolizumab treatment arm was dropped in the phase II redesign of the study

^b^The majority (89%) of non-squamous cell histologies were adenocarcinomas. Other non-squamous histologies included adenosquamous, large cell carcinoma, non-small cell not otherwise specified, poorly differentiated, anaplastic carcinoma, glandular papillary carcinoma, and lymphoepithelioma-like carcinoma

### Treatment duration

Compared with the PBO + P + C group, the E + P + C group had less exposure to study medication, with a lower median number of days on treatment, fewer cycles on treatment, fewer days on epacadostat and fewer patients completing all four initial chemotherapy cycles (Supplementary Table 1). The median duration of follow-up was 5.1 months (range 0.1–10.4) in the E + P + C group and 7.2 months (range 1.4–10.4) in the PBO + P + C group.

### Efficacy

The open-label E + P group was excluded from the efficacy analysis. Based on BICR assessment, superiority of ORR for the E + P + C group compared with the PBO + P + C group could not be claimed (Table 2). The E + P + C group had a lower confirmed ORR (26.4%; 95% confidence interval [CI] 17.7–36.7) than the PBO + P + C group (44.8%; 95% CI 34.1–55.9), with a difference of − 18.5% (95% CI − 32.0 – (− 4.3); one-sided *P* = 0.9948). For PD-L1 TPS < 50%, ORR was 27.8% (95% CI 17.9–39.6) and 40.9% (95% CI 29.0–53.7) in the E + P + C and PBO + P + C groups, respectively. For PD-L1 TPS ≥ 50%, ORR was 21.1% (95% CI 6.1–45.6) and 57.1% (95% CI 34.0–78.2) in the E + P + C and PBO + P + C groups, respectively. No complete responses were observed and the proportion of patients with best response of stable disease was similar in the E + P + C and PBO + P + C groups (41.8%; 95% CI 31.5–52.6 vs. 40.2%; 95% CI 29.9–51.3). The disease control rate was 68.1% (95% CI 57.5–77.5) in the E + P + C group and 85.1% (95% CI 75.8–91.8) in the PBO + P + C group. The open-label E + P treatment arm was terminated by the protocol amendment described above; although efficacy data of the open-label E + P group were not compared with the other two groups, the ORR for this group was 20.0% (95% CI 10.4–33.0).

**Table 2:** Summary of objective response

	Epacadostat + pembrolizumab with chemotherapy (*n* = 91)		Placebo + pembrolizumab with chemotherapy (*n* = 87)	
	*n*	% (95% CI)	*n*	% (95% CI)
CR	0	0 (0.0–4.0)	0	0 (0.0–4.2)
PR	24	26.4 (17.7–36.7)	39	44.8 (34.1–55.9)
Overall response^a^	24	26.4 (17.7–36.7)	39	44.8 (34.1–55.9)
SD^b^	38	41.8 (31.5–52.6)	35	40.2 (29.9–51.3)
Disease control^c^	62	68.1 (57.5–77.5)	74	85.1 (75.8–91.8)
PD	14	15.4 (8.7–24.5)	8	9.2 (4.1–17.3)
NE^d^	7	7.7 (3.1–15.2)	2	2.3 (0.3–8.1)
No assessment^e^	8	8.8 (3.9–16.6)	3	3.4 (0.7–9.7)
Patients with a response^f^	24		39	
TTR, median (range), months	1.6 (1.2–6.1)		1.4 (1.1–6.2)	
DOR,^g^ median (range), months	NR (1.1 + to 7.0 +)		7.0 (1.2 + to 8.0 +)	
Patients with ongoing response, *n* (%)^h^	20 (83.3)		29 (74.4)	
≥ 3 months	14 (58.3)		19 (48.7)	
≥ 6 months	7 (29.2)		6 (15.4)	

Responses based on BICR assessment per RECIST v1.1

*BICR* Blinded independent central review, *CI* Confidence interval, *CR* Complete response, *DOR* Duration of response, *NE* Not evaluable, *NR* Not reached, *PD* Progressive disease, *PR* Partial response, *RECIST v1.1* Response Evaluation Criteria In Solid Tumors version 1.1, *SD* Stable disease, *TTR* time to response

^a^Overall response includes CR and PR

^b^SD includes both SD and Non-CR/Non-PD

^c^Disease control includes CR, PR and SD

^d^Post-baseline assessment(s) available but not evaluable or CR/PR/SD < 6 weeks from randomization

^e^No post-baseline assessment available

^f^Includes patients with best objective response as confirmed CR or PR

^g^From product-limit (Kaplan–Meier) method for censored data. " + " indicates there is no PD by the time of last disease assessment

^h^Includes patients who are alive, have not progressed, have not initiated new anticancer treatment, are not lost to follow-up and whose last disease assessment was < 5 months prior to data cutoff date

Because the prespecified success criterion for the primary ORR hypothesis was not met, the study was unblinded, all epacadostat and placebo administration was stopped (all remaining patients had continued access to pembrolizumab), and the subsequent efficacy hypotheses were not formally tested. The median DOR was not reached (range 1.1 + to 7.0 +) in the E + P + C group and was 7.0 months (range 1.2 + to 8.0 +) in the PBO + P + C group (plus symbols [ +] indicate no progressive disease by the time of last disease assessment); the median TTR was similar for the E + P + C and PBO + P + C groups (Table 2). Among confirmed responders, similar proportions of patients had ongoing responses in both groups. However, the E + P + C group had a numerically higher percentage of confirmed responders with an extended response ≥ 6 months (29.2% vs. 15.4%).

**Fig. 2:**
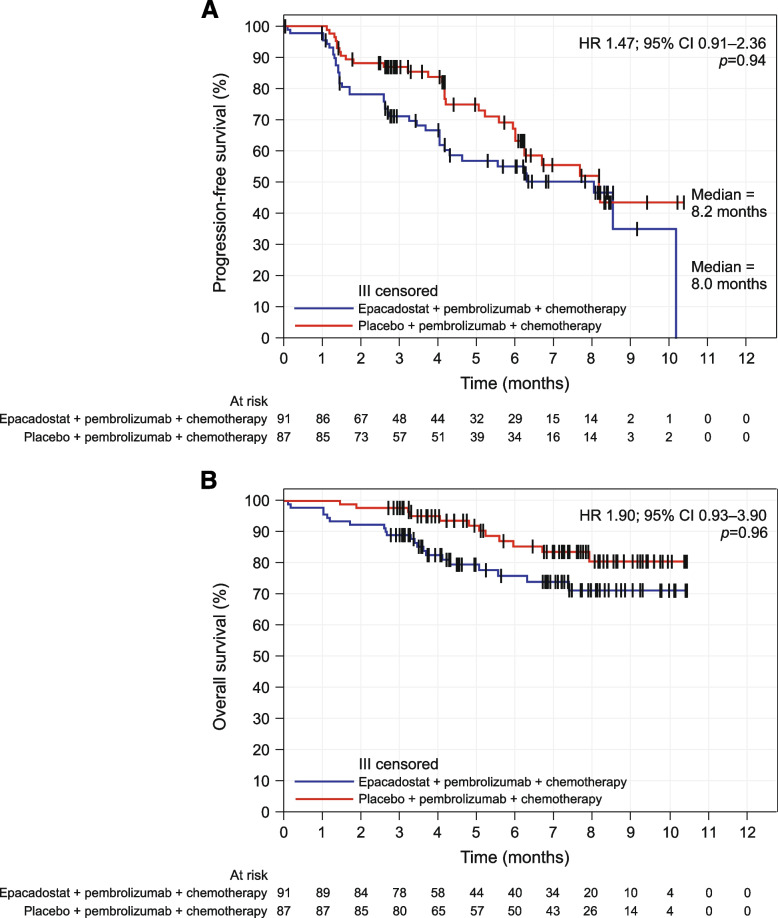
Kaplan–Meier curves for **a** PFS^a^ and **b** OS. ^a^Based on BICR assessment per RECIST v1.1. One-sided p-value based on log-rank test stratified by PD-L1 TPS (< 50% vs ≥ 50%) and predominant tumor histology (squamous vs non-squamous), because of small sample size, the strata ‘PD-L1 TPS greater than or equal to 50 percent Non-squamous’ and ‘PD-L1 TPS greater than or equal to 50 percent. Squamous’ were combined into one stratum. *BICR* blinded independent central review; *OS* overall survival; *PFS* progression-free survival; *RECIST v1.1* Response Evaluation Criteria in Solid Tumors version 1.1

The median PFS was 8.0 months for the E + P + C group and 8.2 months for the PBO + P + C group (HR 1.47; 95% CI 0.91–2.36) (Fig. 2A). The PFS rates at 3, 6 and 9 months were numerically lower in the E + P + C versus the PBO + P + C group. The median OS was not reached in either group (Fig. 2B). The OS rates were numerically lower in the E + P + C versus the PBO + P + C group at 3, 6 and 9 months.

A subgroup analysis did not identify any baseline characteristics associated with improved responses (Supplementary Table 2). Subgroups included age, baseline ECOG status, gender, smoking status, baseline brain metastasis, PD-L1 TPS (≥ 50% or < 50%), investigator choice of chemotherapy, predominant tumor histology, race (white vs. non-white), metastatic stage and non-East Asian geographic region.

### Safety and tolerability

The frequency of AEs, drug-related AEs, grade 3 − 5 AEs, drug-related grade 3 − 5 AEs, serious AEs (SAEs) and drug-related SAEs were slightly higher (< 10% difference) in the E + P + C group compared with the PBO + P + C group (Table 3). Anemia was less frequent in the E + P + C group (24.4%) than in the PBO + P + C group (38.4%). In the E + P + C group, vomiting (22.2%) and rash (25.6%) were more frequent than in the PBO + P + C group (vomiting, 9.3%; rash, 18.6%). The most frequent SAEs (> 2 patients) in the E + P + C group were febrile neutropenia (4.4%), pneumonia (4.4%) and lower respiratory tract infection (3.3%). Of these, only pneumonia was reported in the PBO + P + C group (12.8%). The most frequent (> 2%) drug-related SAEs reported in the E + P + C group were febrile neutropenia (4.4%), diarrhea (2.2%), lower respiratory tract infection (2.2%) and neutropenia (2.2%). Of these, only diarrhea was reported in the PBO + P + C group at a similar rate (2.3%). The most frequent drug-related SAE in the PBO + P + C group, pneumonia (3.5%), was not reported in the E + P + C group. No serotonin syndrome related to epacadostat was observed in this trial.

**Table 3:** Summary of adverse events

	Epacadostat + pembrolizumab with chemotherapy (*n* = 90)		Placebo + pembrolizumab with chemotherapy (*n* = 86)		Epacadostat + pembrolizumab (*n* = 52)	
	Any AE	Drug-related AE	Any AE	Drug-related AE	Any AE	Drug-related AE
Any AE^a^	89 (98.9)	85 (94.4)^b^	82 (95.3)	77 (89.5)^b^	51 (98.1)	40 (76.9)^b^
Nausea	36 (40.0)	35 (38.9)	36 (41.9)	31 (36.0)	10 (19.2)	6 (11.5)
Fatigue	26 (28.9)	21 (23.3)	23 (26.7)	20 (23.3)	12 (23.1)	10 (19.2)
Constipation	25 (27.8)	11 (12.2)	22 (25.6)	7 (8.1)	7 (13.5)	0
Rash	23 (25.6)	21 (23.3)	16 (18.6)	13 (15.1)	8 (15.4)	6 (11.5)
Anemia	22 (24.4)	19 (21.1)	33 (38.4)	26 (30.2)	8 (15.4)	2 (3.8)
Diarrhea	20 (22.2)	15 (16.7)	20 (23.3)	14 (16.3)	11 (21.2)	7 (13.5)
Vomiting	20 (22.2)	12 (13.3)	8 (9.3)	5 (5.8)	5 (9.6)	2 (3.8)
Grade 3–5 AE	58 (64.4)	42 (46.7)	52 (60.5)	36 (41.9)	23 (44.2)	11 (21.2)
SAE	39 (43.3)	23 (25.6)	32 (37.2)	16 (18.6)	12 (23.1)	3 (5.8)
Dose modification^c^ due to an AE	62 (68.9)	–	52 (60.5)	–	24 (46.2)	–
Pembrolizumab	47 (52.2)	–	40 (46.5)	–	18 (34.6)	–
Epacadostat/placebo	56 (62.2)	–	47 (54.7)	–	22 (42.3)	–
Carboplatin	36 (40.0)	–	27 (31.4)	–	0	–
Cisplatin	2 (2.2)	–	1 (1.2)	–	0	–
Paclitaxel	6 (6.7)	–	10 (11.6)	–	0	–
Pemetrexed	44 (48.9)	–	33 (38.4)	–	0	–
Discontinued due to an AE	23 (25.6)	18 (20.0)	21 (24.4)	16 (18.6)	5 (9.6)	4 (7.7)
Pembrolizumab	15 (16.7)	10 (11.1)	14 (16.3)	9 (10.5)	4 (7.7)	3 (5.8)
Epacadostat/placebo	18 (20.0)	13 (14.4)	17 (19.8)	12 (14.0)	5 (9.6)	4 (7.7)
Carboplatin	9 (10.0)	6 (6.7)	1 (1.2)	0	0	0
Cisplatin	2 (2.2)	1 (1.1)	0	0	0	0
Paclitaxel	2 (2.2)	2 (2.2)	0	0	0	0
Pemetrexed	15 (16.7)	10 (11.1)	10 (11.6)	7 (8.1)	0	0
Deaths	5 (5.6)	2 (2.2)	2 (2.3)	0	2 (3.8)	0

Data are *n* (%)

Non-serious AEs up to 30 days of last dose and SAEs up to 90 days of last dose are included

MedDRA preferred terms "Neoplasm progression", "Malignant neoplasm progression" and "Disease progression" not related to the drug are excluded

Grades are based on NCI CTCAE version 4.0

^a^AEs (any grade) in ≥ 20% of patients in the E + P + C or PBO + P + C treatment arms

^b^Determined by the investigator to be related to the drug

^c^Defined as an action taken of dose reduced, drug interrupted, or drug withdrawn

– not reported; *AE* adverse event; *NCI CTCAE* National Cancer Institute Common Terminology Criteria for Adverse Events; *SAE* serious adverse event

A higher percentage of patients in the E + P + C group had dose modifications of study drugs due to an AE compared with the PBO + P + C group (Table 3). Specifically, dose modification of pembrolizumab, epacadostat/placebo, carboplatin and pemetrexed were more frequent in the E + P + C group versus the PBO + P + C group. Similar proportions of patients in the E + P + C and PBO + P + C groups discontinued one or more study interventions due to drug-related AEs/SAEs (Table 3). Discontinuations of pembrolizumab or epacadostat/placebo were similar in both groups, while discontinuations of chemotherapy were more frequent in the E + P + C group versus the PBO + P + C group. Two deaths due to drug-related AEs (pneumonitis and sepsis) were reported in the E + P + C group and none in the PBO + P + C group (Table 3).

### Pharmacodynamic activity of epacadostat

**Fig. 3:**
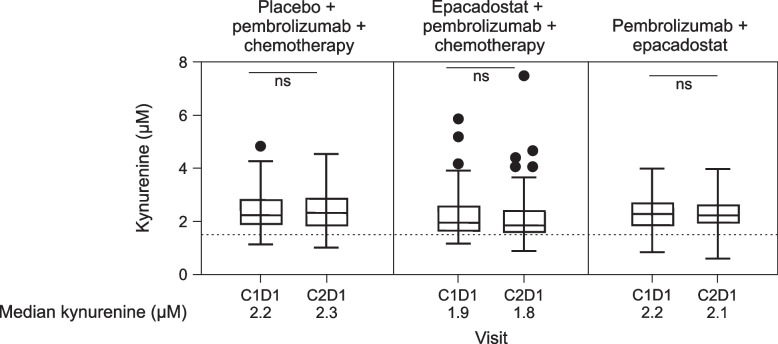
Circulating kynurenine levels at baseline (C1D1) and after one cycle of treatment (C2D1). The number of samples assessed was 83 in the placebo plus pembrolizumab and chemotherapy group, 74 in the epacadostat plus pembrolizumab and chemotherapy group (73 for C1), and 48 in the pembrolizumab plus epacadostat group. C1D1 vs. C2D1 were compared within each treatment arm using paired t-tests. Vertical lines represent maximum and minimum values, horizontal lines represent the median values, bars represent the interquartile range (25th-75th) percentiles, and dots represent outlier values. The dotted line indicates the median kynurenine levels in healthy subjects (1.5 μM) [16]. *C* cycle; *D* day; *ns* not significant

Median circulating kynurenine levels were measured at baseline (C1D1) and after one cycle of treatment (C2D1) (Fig. 3). In all treatment arms, circulating kynurenine levels were similar at C1D1 and C2D1 (PBO + P + C: 2.2 µM vs. 2.3 µM; E + P + C: 1.9 µM vs. 1.8 µM; E + P: 2.2 µM vs. 2.1 µM; all *P* = NS) and above the median levels observed in healthy subjects (1.5 μM) [16].

## Discussion

In ECHO-306/KEYNOTE-715, the addition of epacadostat to pembrolizumab + chemotherapy did not meet the prespecified success criterion for the primary ORR hypothesis in patients with previously untreated metastatic NSCLC. Subgroup analyses did not suggest any patient subgroup who experienced an improved response. PFS and OS differences between treatment arms were not conclusive to confirm an effect of the combination of epacadostat + pembrolizumab + chemotherapy. The median PFS and ORR in the placebo arm were consistent with those reported in other pembrolizumab + chemotherapy NSCLC studies [4, 20]. The addition of epacadostat to pembrolizumab + chemotherapy was generally well tolerated with a safety profile consistent with those previously observed for epacadostat and pembrolizumab + chemotherapy. No new AEs of safety concern were identified, but more patients in the epacadostat group discontinued chemotherapy.

In the current study, the addition of epacadostat to pembrolizumab plus chemotherapy did not provide additional benefit based on ORR, and the ORR was lower in the E + P + C arm. We cannot discount the possibility that epacadostat had a negative impact on outcomes. Although this trend is potentially of concern, it has not been consistently seen in other trials of epacadostat combinations (see accompanying articles, *this issue*). Also, given the lack of pharmacodynamic effect based on peripheral blood kynurenine analysis, there is no obvious biologic reason for why epacadostat 100 mg BID would reduce the quality of response to PD-1 inhibition in combination with chemotherapy. It also is unlikely that levels of epacadostat were altered due to drug-drug interactions. In a separate study (ECHO-207/KEYNOTE-723), no effects of pembrolizumab with various chemotherapies on epacadostat exposure were observed [21]. Instead, the reduction in ORR might have been the result of the differences in tolerability and safety observed with epacadostat versus placebo, imbalance between treatment arms in baseline prognostic factors, or the result of chance. Patients in the epacadostat arm had lower exposure to study drugs than those in the placebo arm as shown by more frequent dose modification of study drugs due to an AE, higher chemotherapy discontinuation rates (specifically, for carboplatin or pemetrexed) and fewer days and cycles of study treatments. In addition, there was a higher percentage of patients with stage M1C tumors at baseline in the epacadostat treatment group. The E + P + C arm also had a slightly higher percentage of patients with squamous NSLC histology, which is known to have a poorer prognosis than non-squamous NSCLC [4, 20, 22].

Studies that assessed addition of epacadostat to pembrolizumab for other types of cancers also did not meet prespecified efficacy endpoints. In the phase III ECHO-301/KEYNOTE-252 study evaluating epacadostat plus pembrolizumab versus placebo plus pembrolizumab in metastatic melanoma, the primary endpoint of PFS was not met [18]. Other reports in this supplement present similar findings in NSCLC (PD-L1 TPS ≥ 50%), urothelial carcinoma, renal cell carcinoma and head and neck squamous cell carcinoma (see accompanying articles, *this issue*).

Epacadostat 100 mg BID was chosen for evaluation in this and other studies on the basis of a potential well-tolerated safety profile in combination with pembrolizumab and chemotherapy, robust ORRs, durable disease control rates, and the observation that epacadostat monotherapy at BID doses ≥ 100 mg has been shown to normalize plasma kynurenine levels in patients with advanced solid tumors [16]. However, in this study, epacadostat 100 mg BID did not appear to reduce the levels of circulating kynurenine in patients with NSCLC when administered with pembrolizumab plus chemotherapy or pembrolizumab alone. Although not directly observed in the PBO + P + C arm of the current study, pembrolizumab has been reported to enhance kynurenine production [23]. Furthermore, a retrospective analysis of epacadostat clinical studies showed that epacadostat doses of ≥ 600 mg BID may be needed to normalize plasma and intratumoral kynurenine levels when given in combination with a checkpoint inhibitor [23]. Thus, when used in combination with pembrolizumab, higher doses of epacadostat may be needed to normalize kynurenine levels. Evaluation of higher epacadostat doses may shed light on the potential efficacy of combination treatment with epacadostat and pembrolizumab.

Other studies are also evaluating the combination of IDO1 and PD-1/PD-L1 inhibition for the treatment of cancer. Epacadostat plus the PD-1 inhibitor nivolumab is being assessed in patients with advanced solid tumors, including NSCLC, in the phase I/II ECHO-204 study; promising preliminary antitumor activity has been reported, particularly in squamous cell carcinoma of the head and neck with epacadostat 300 mg BID in combination with nivolumab and in melanoma with epacadostat 100 mg BID or 300 mg BID in combination with nivolumab [24]. A phase I trial assessing the IDO1 inhibitor navoximod with the PD-L1 inhibitor atezolizumab showed antitumor activity in various tumor types, including NSCLC; however, no clear benefit of adding navoximod was seen [25]. Identification of biomarkers associated with a benefit of combined IDO1 and PD-1/PD-L1 inhibition is needed to determine the usefulness of this approach in specific patient populations and to guide further studies.

Limitations of our study include the small sample size, the change in study design from phase III to phase II during the study and the early study discontinuation. Because ORR is not a validated surrogate endpoint for OS with checkpoint inhibitors, this study did not directly assess the potential clinical benefit. Subgroup analyses based on epacadostat pharmacodynamics was not possible due to the small fraction of patients with reduced serum kynurenine.

## Conclusions

The addition of epacadostat 100 mg BID to pembrolizumab and platinum-based chemotherapy was generally well tolerated, but this study did not meet the pre-specified success criterion for the primary ORR hypothesis in this population of frontline patients with NSCLC. Additional biomarker analyses and clinical studies are needed to determine if a combination strategy including epacadostat (possibly at higher doses) could provide clinical benefit in selected populations.

### Supplementary Information

**Additional file 1:** **Supplementary Table 1.** Exposure to study medication.

**Additional file 2:** **Supplementary Table 2.** Subgroup analysis of objective responses based on blinded independent central review (ITT population).

## Data Availability

Access to individual patient-level data is not available for this study.

## Abbreviations

AE: Adverse events
AUC: Area under the curve
BICR: Blinded independent central review
BID: Twice daily
C#D#: Cycle # day #
CI: Confidence interval
CNS: Central nervous system
CR: Complete response
CTCAE: Common Terminology Criteria for Adverse Events
DOR: Duration of response
E: Epacadostat
ECOG: Eastern Cooperative Oncology Group
HR: Hazard ratio
IDO1: Indoleamine 2,3-dioxygenase 1
IV: Intravenous
NCI: National Cancer Institute
NE: Not evaluable
NR: Not reached
NSCLC: Non-small cell lung cancer
ORR: Objective response rate
OS: Overall survival
P: Pembrolizumab
PBO: Placebo
PD: Progressive disease
PD-1: Programmed cell death protein 1
PD-L1: Programmed death-ligand 1
PFS: Progression-free survival
PR: Partial response
Q3W: Every 3 weeks (21 days)
RECIST v1.1: Response Evaluation Criteria In Solid Tumors version 1.1
SAE: Serious adverse event
SD: Stable disease (Table 1) or Standard deviation (Table 2)
TPS: Tumor proportion score
TTR: Time to response

## References

[1] American Cancer Society. Cancer Facts & Figures 2023. Atlanta: American Cancer Society; 2023.

[2] Besse B, Adjei A, Baas P, Melgaard P, Nicolson M, Paz-Ars L, et al. 2nd ESMO Consensus Conference on Lung Cancer: non-small-cell lung cancer first-line/second and further lines of treatment in advanced disease. Ann Oncol. 2014;25(8):1475–84. 24669016 10.1093/annonc/mdu123

[3] Chen Y, Zhou Y, Tang L, Peng X, Jiang H, Wang G, et al. Immune-checkpoint inhibitors as the first line treatment of advanced non-small cell lung cancer: a meta-analysis of randomized controlled trials. J Cancer. 2019;10(25):6261–8. 31772659 10.7150/jca.34677 PMC6856743

[4] Gandhi L, Rodriguez-Abreu D, Gadgeel S, Esteban E, Felip E, De Angelis F, et al. Pembrolizumab plus chemotherapy in metastatic non-small-cell lung cancer. N Engl J Med. 2018;378(22):2078–92. 29658856 10.1056/NEJMoa1801005

[5] Mellor AL, Munn DH. IDO expression by dendritic cells: tolerance and tryptophan catabolism. Nat Rev Immunol. 2004;4(10):762–74. 15459668 10.1038/nri1457

[6] Uyttenhove C, Pilotte L, Theate I, Stroobant V, Colau D, Parmentier N, et al. Evidence for a tumoral immune resistance mechanism based on tryptophan degradation by indoleamine 2,3-dioxygenase. Nat Med. 2003;9(10):1269–74. 14502282 10.1038/nm934

[7] Schalper KA, Carvajal-hausdorf D, McLaughlin J, Altan M, Velcheti V, Gaule P, et al. Differential expression and significance of PD-L1, IDO-1, and B7–H4 in human lung cancer. Clin Cancer Res. 2017;23(2):370–8. 27440266 10.1158/1078-0432.CCR-16-0150 PMC6350535

[8] Takada K, Kohashi K, Shimokawa M, Haro A, Osoegawa A, Tagawa T, et al. Co-expression of IDO1 and PD-L1 in lung squamous cell carcinoma: Potential targets of novel combination therapy. Lung Cancer. 2019;128:26–32. 30642449 10.1016/j.lungcan.2018.12.008

[9] Volaric A, Gentzler R, Hall R, Mehaffey JH, Stelow EB, Bullock TN, et al. Indoleamine-2,3-dioxygenase in non-small cell lung cancer: a targetable mechanism of immune resistance frequently coexpressed with PD-L1. Am J Surg Pathol. 2018;42(9):1216–23. 29901571 10.1097/PAS.0000000000001099

[10] Fallarino F, Grohmann U, You S, McGrath BC, Cavener DR, Vacca C, et al. The combined effects of tryptophan starvation and tryptophan catabolites down-regulate T cell receptor zeta-chain and induce a regulatory phenotype in naive T cells. J Immunol. 2006;176(11):6752–61. 16709834 10.4049/jimmunol.176.11.6752

[11] Botticelli A, Cerbelli B, Lionetto L, Zizzari I, Salati M, Pisano A, et al. Can IDO activity predict primary resistance to anti-PD-1 treatment in NSCLC? J Transl Med. 2018;16(1):219. 30081936 10.1186/s12967-018-1595-3 PMC6080500

[12] Holmgaard RB, Zamarin D, Munn DH, Wolchok JD, Allison JP. Indoleamine 2,3-dioxygenase is a critical resistance mechanism in antitumor T cell immunotherapy targeting CTLA-4. J Exp Med. 2013;210(7):1389–402. 23752227 10.1084/jem.20130066 PMC3698523

[13] Taylor MW, Feng GS. Relationship between interferon-gamma, indoleamine 2,3-dioxygenase, and tryptophan catabolism. FASEB J. 1991;5(11):2516–22. 1907934 10.1096/fasebj.5.11.1907934

[14] Toulmonde M, Penel N, Adam J, Chevreau C, Blay J-Y, Le Cesne A, et al. Use of PD-1 targeting, macrophage infiltration, and IDO pathway activation in sarcomas: a phase 2 clinical trial. JAMA Oncol. 2018;4(1):93–7. 28662235 10.1001/jamaoncol.2017.1617 PMC5833654

[15] Liu X, Shin N, Koblish HK, Yang G, Wang Q, Wang K, et al. Selective inhibition of IDO1 effectively regulates mediators of antitumor immunity. Blood. 2010;115(17):3520–30. 20197554 10.1182/blood-2009-09-246124

[16] Beatty GL, O’Dwyer PJ, Clark J, Shi JG, Bowman KJ, Scherle PA, et al. First-in-human phase I study of the oral inhibitor of indoleamine 2,3-dioxygenase-1 epacadostat (INCB024360) in patients with advanced solid malignancies. Clin Cancer Res. 2017;23(13):3269–76. 28053021 10.1158/1078-0432.CCR-16-2272 PMC5496788

[17] ClinicalTrials.gov. Study to explore the safety, tolerability and efficacy of MK-3475 in combination with INCB024360 in participants with selected cancers. Identifier NCT02178722.

[18] Long GV, Dummer R, Hamid O, Gajewski TF, Caglevic C, Dalle S, et al. Epacadostat plus pembrolizumab versus placebo plus pembrolizumab in patients with unresectable or metastatic melanoma (ECHO-301/KEYNOTE-252): a phase 3, randomised, double-blind study. Lancet Oncol. 2019;20(8):1083–97. 31221619 10.1016/S1470-2045(19)30274-8

[19] Miettinen O, Nurminen M. Comparative analysis of two rates. Stat Med. 1985;4(2):213–26. 4023479 10.1002/sim.4780040211

[20] Paz-Ares L, Luft A, Vicente D, Tafreshi A, Gümüş M, Mazières J, et al. Pembrolizumab plus Chemotherapy for Squamous Non-Small-Cell Lung Cancer. N Engl J Med. 2018;379(21):2040–51. 30280635 10.1056/NEJMoa1810865

[21] Powderly JD, Klempner SJ, Naing A, Bendell J, Garrido-Laguna I, Catenacci DVT, et al. Epacadostat Plus Pembrolizumab and Chemotherapy for Advanced Solid Tumors: Results from the Phase I/II ECHO-207/KEYNOTE-723 Study. Oncologist. 2022;27(11):905-e848. 10.1093/oncolo/oyac174 PMC9632315 36156099

[22] Mok TSK, Wu Y-L, Kudaba I, Kowalski DM, Cho BC, et al. Pembrolizumab versus chemotherapy for previously untreated, PD-L1-expressing, locally advanced or metastatic non-small-cell lung cancer (KEYNOTE-042): a randomised, open-label, controlled, Phase 3 trial. Lancet. 2019;393:1819–30. 30955977 10.1016/S0140-6736(18)32409-7

[23] Smith M, Newton R, Owens S, Gong X, Tian C, Maleski J, et al. Retrospective pooled analysis of epacadostat clinical studies identifies doses required for maximal pharmacodynamic effect in anti-PD-1 combination studies. J Immunother Cancer. 2020;8(Suppl 3):A15-6.

[24] Perez RP, Reis MJ, Lewis KD, Saleh MN, Daud A, Berlin J, et al. Epacadostat plus nivolumab in patients with advanced solid tumors: Preliminary phase I/II results of ECHO-204. J Clin Oncol. 2017;35(suppl 15):3003. 10.1200/JCO.2017.35.15_suppl.3003

[25] Jung KH, LoRusso P, Burris H, Gordon M, Bang YJ, Hellmann MD, et al. Phase I study of the indoleamine 2,3-dioxygenase 1 (IDO1) inhibitor navoximod (GDC-0919) administered with PD-L1 inhibitor (atezolizumab) in advanced solid tumors. Clin Cancer Res. 2019;25(11):3220–8. 30770348 10.1158/1078-0432.CCR-18-2740 PMC7980952

