# Supplementary material for: Pembrolizumab with platinum-based chemotherapy with or without epacadostat as first-line treatment for metastatic non-small cell lung cancer: a randomized, partially double-blind, placebo-controlled phase II study
Source: BMC Cancer. 2024 Jul 25;23(Suppl 1):1250. doi: 10.1186/s12885-022-10427-4 (PMC11270757; doi:10.1186/s12885-022-10427-4)
Supplement: Supplementary file 1 — Additional file 1: Supplementary Table 1. Exposure to study medication. [file 12885_2022_10427_MOESM1_ESM.docx]

**Supplementary Table 1** Exposure to study medication

|  | Epacadostat + pembrolizumab with chemotherapy  (*n* = 90) | Placebo + pembrolizumab with chemotherapy  (*n* = 86) | Epacadostat + pembrolizumab  (*n* = 52) |
| --- | --- | --- | --- |
| Number of days on treatment | 126.0 (2.0–341.0) | 163.0 (8.0–341.0) | 164.5 (6.0–303.0) |
| Number of days on epacadostat/placebo | 107.0 (2.0–341.0) | 148.0 (8.0–341.0) | 150.5 (6.0–303.0) |
| Number of cycles of treatment | 6.0 (1.0–16.0) | 8.0 (1.0–15.0) | 7.0 (1.0–14.0) |
| Number of days on pembrolizumab | 106.0 (1.0–316.0) | 150.5 (1.0–314.0) | 154.0 (1.0–283.0) |
| All data are median (range) | | | |
