# Supplementary material for: Pembrolizumab with platinum-based chemotherapy with or without epacadostat as first-line treatment for metastatic non-small cell lung cancer: a randomized, partially double-blind, placebo-controlled phase II study
Source: BMC Cancer. 2024 Jul 25;23(Suppl 1):1250. doi: 10.1186/s12885-022-10427-4 (PMC11270757; doi:10.1186/s12885-022-10427-4)
Supplement: Supplementary file 2 — Additional file 2: Supplementary Table 2. Subgroup analysis of objective responses based on blinded independent central review (ITT population). [file 12885_2022_10427_MOESM2_ESM.docx]

**Supplementary Table 2.** Subgroup analysis of objective responses based on blinded independent central review (ITT population).

|  | Pembro + Epacadostat + Chemo | | | | Pembro + Chemo | | | |  | |
| --- | --- | --- | --- | --- | --- | --- | --- | --- | --- | --- |
|  | (N=91) | | | | (N=87) | | | | Difference^†^ | |
|  | N | n | (%) | 95% CI (%) | N | n | (%) | 95% CI (%) | (%) | 95% CI (%) |
| Overall | 91 | 24 | (26.4) | (17.7, 36.7) | 87 | 39 | (44.8) | (34.1, 55.9) | (-18.5) | (-32.0, -4.3) |
| **PD-L1 TPS** | | | | | | | | | | |
| < 50% | 72 | 20 | (27.8) | (17.9, 39.6) | 66 | 27 | (40.9) | (29.0, 53.7) | (-13.1) | (-28.6, 2.8) |
| ≥ 50% | 19 | 4 | (21.1) | (6.1, 45.6) | 21 | 12 | (57.1) | (34.0, 78.2) | (-36.1) | (-60.5, -5.4) |
| **Predominant Tumor Histology** | | | | | | | | | | |
| Squamous | 26 | 7 | (26.9) | (11.6, 47.8) | 22 | 13 | (59.1) | (36.4, 79.3) | (-32.2) | (-56.0, -3.9) |
| Non Squamous | 65 | 17 | (26.2) | (16.0, 38.5) | 65 | 26 | (40.0) | (28.0, 52.9) | (-13.8) | (-29.5, 2.4) |
| **Age (Years)** | | | | | | | | | | |
| < 65 | 49 | 16 | (32.7) | (19.9, 47.5) | 46 | 20 | (43.5) | (28.9, 58.9) | (-10.8) | (-29.7, 8.7) |
| ≥ 65 | 42 | 8 | (19.0) | (8.6, 34.1) | 41 | 19 | (46.3) | (30.7, 62.6) | (-27.3) | (-45.6, -7.2) |
| **Sex** | | | | | | | | | | |
| Male | 58 | 13 | (22.4) | (12.5, 35.3) | 57 | 25 | (43.9) | (30.7, 57.6) | (-21.4) | (-37.6, -4.2) |
| Female | 33 | 11 | (33.3) | (18.0, 51.8) | 30 | 14 | (46.7) | (28.3, 65.7) | (-13.3) | (-36.3, 10.9) |
| **Race** | | | | | | | | | | |
| White | 78 | 21 | (26.9) | (17.5, 38.2) | 75 | 31 | (41.3) | (30.1, 53.3) | (-14.4) | (-29.0, 0.7) |
| Non-White | 13 | 3 | (23.1) | (5.0, 53.8) | 11 | 8 | (72.7) | (39.0, 94.0) | (-49.7) | (-76.2, -8.9) |
| **Baseline** **ECOG** | | | | | | | | | | |
| 0 | 35 | 8 | (22.9) | (10.4, 40.1) | 27 | 12 | (44.4) | (25.5, 64.7) | (-21.6) | (-43.9, 2.1) |
| 1 | 55 | 16 | (29.1) | (17.6, 42.9) | 60 | 27 | (45.0) | (32.1, 58.4) | (-15.9) | (-32.7, 1.9) |

Overall response based on best overall response using BICR assessment per RECIST 1.1 with confirmation.

^†^ Analysis (ORR difference and 95% CI) for the overall population and the PD-L1 subgroups is based on the stratified Miettinen & Nurminen method; analysis for the other subgroups is based on the unstratified Miettinen & Nurminen method.

**Supplementary Table 2 *continued*. Subgroup analysis of objective responses based on blinded independent central review (ITT population).**

|  | Pembro + Epacadostat + Chemo | | | | Pembro + Chemo | | | |  | |
| --- | --- | --- | --- | --- | --- | --- | --- | --- | --- | --- |
|  | (N=91) | | | | (N=87) | | | | Difference^†^ | |
|  | N | n | (%) | 95% CI (%) | N | n | (%) | 95% CI (%) | (%) | 95% CI (%) |
| Overall | 91 | 24 | (26.4) | (17.7, 36.7) | 87 | 39 | (44.8) | (34.1, 55.9) | (-18.5) | (-32.0, -4.3) |
| **Geographic Region** | | | | | | | | | | |
| Non-East Asia | 82 | 22 | (26.8) | (17.6, 37.8) | 81 | 34 | (42.0) | (31.1, 53.5) | (-15.1) | (-29.2, -0.5) |
| **Smoking Status** | | | | | | | | | | |
| Never Smoker | 17 | 3 | (17.6) | (3.8, 43.4) | 12 | 5 | (41.7) | (15.2, 72.3) | (-24.0) | (-55.1, 9.4) |
| Former/Current Smoker | 74 | 21 | (28.4) | (18.5, 40.1) | 75 | 34 | (45.3) | (33.8, 57.3) | (-17.0) | (-31.7, -1.4) |
| **History of Brain Metastasis** | | | | | | | | | | |
| Yes | 10 | 1 | (10.0) | (0.3, 44.5) | 11 | 2 | (18.2) | (2.3, 51.8) | (-8.2) | (-41.2, 27.1) |
| No | 81 | 23 | (28.4) | (18.9, 39.5) | 76 | 37 | (48.7) | (37.0, 60.4) | (-20.3) | (-34.7, -5.1) |
| **Investigator’s Choice of Chemotherapy** | | | | | | | | | | |
| Carboplatin/ Paclitaxel | 27 | 7 | (25.9) | (11.1, 46.3) | 23 | 13 | (56.5) | (34.5, 76.8) | (-30.6) | (-54.2, -3.1) |
| Carboplatin/ Pemetrexed | 57 | 16 | (28.1) | (17.0, 41.5) | 56 | 23 | (41.1) | (28.1, 55.0) | (-13.0) | (-29.9, 4.6) |
| **Metastatic Stage** | | | | | | | | | | |
| M1A | 30 | 6 | (20.0) | (7.7, 38.6) | 29 | 15 | (51.7) | (32.5, 70.6) | (-31.7) | (-52.9, -7.2) |
| M1B | 15 | 6 | (40.0) | (16.3, 67.7) | 25 | 13 | (52.0) | (31.3, 72.2) | (-12.0) | (-40.9, 19.8) |
| M1C | 45 | 12 | (26.7) | (14.6, 41.9) | 33 | 11 | (33.3) | (18.0, 51.8) | (-6.7) | (-27.5, 13.6) |

Overall response based on best overall response using BICR assessment per RECIST 1.1 with confirmation.

^†^ Analysis (ORR difference and 95% CI) for the overall population and the PD-L1 subgroups is based on the stratified Miettinen & Nurminen method; analysis for the other subgroups is based on the unstratified Miettinen & Nurminen method.
